# Gene flow during glacial habitat shifts facilitates character displacement in a Neotropical flycatcher radiation

**DOI:** 10.1186/s12862-017-1047-3

**Published:** 2017-09-01

**Authors:** Balaji Chattopadhyay, Kritika M. Garg, Chyi Yin Gwee, Scott V. Edwards, Frank E. Rheindt

**Affiliations:** 1 Department of Biological Sciences, National University of Singapore, 117543 Singapore, Republic of Singapore; 2 Department of Organismic and Evolutionary Biology, Harvard University, Cambridge, 02138 MA USA

**Keywords:** *albiceps* complex, Ice age, MP-EST, *BEAST, Fastsimcoal

## Abstract

**Background:**

Pleistocene climatic fluctuations are known to be an engine of biotic diversification at higher latitudes, but their impact on highly diverse tropical areas such as the Andes remains less well-documented. Specifically, while periods of global cooling may have led to fragmentation and differentiation at colder latitudes, they may – at the same time – have led to connectivity among insular patches of montane tropical habitat with unknown consequences on diversification. In the present study we utilized ~5.5 kb of DNA sequence data from eight nuclear loci and one mitochondrial gene alongside diagnostic morphological and bioacoustic markers to test the effects of Pleistocene climatic fluctuations on diversification in a complex of Andean tyrant-flycatchers of the genus *Elaenia*.

**Results:**

Population genetic and phylogenetic approaches coupled with coalescent simulations demonstrated disparate levels of gene flow between the taxon *chilensis* and two parapatric *Elaenia* taxa predominantly during the last glacial period but not thereafter, possibly on account of downward shifts of montane forest habitat linking the populations of adjacent ridges. Additionally, morphological and bioacoustic analyses revealed a distinct pattern of character displacement in coloration and vocal traits between the two sympatric taxa *albiceps* and *pallatangae*, which were characterized by a lack of gene flow.

**Conclusion:**

Our study demonstrates that global periods of cooling are likely to have facilitated gene flow among Andean montane *Elaenia* flycatchers that are more isolated from one another during warm interglacial periods such as the present era. We also identify a hitherto overlooked case of plumage and vocal character displacement, underpinning the complexities of gene flow patterns caused by Pleistocene climate change across the Andes.

**Electronic supplementary material:**

The online version of this article (doi:10.1186/s12862-017-1047-3) contains supplementary material, which is available to authorized users.

## Background

Evolutionary processes of diversification rely on environmental change to bring about shifts in the distributions of habitats and species. One of the greatest planetary engines of environmental change driving biotic diversification over the last 2,000,000 years has been the cyclical succession of periods of global cooling, the so called ‘ice ages’ [1–3]. Over the Quaternary period, ice ages have regularly led to significant earth-historic change through fluctuations in temperature, sea level and extensive glaciations.

The effects of ice ages on the biota of the northern hemispheric temperate regions are relatively well understood [4–8]. But, despite the wealth of research that has gone into the investigation of the effects of Pleistocene glaciations on biotic differentiation in northern temperate latitudes, much less is known about how periods of global cooling have impacted the most species-rich tropical regions of the world (for exceptions see, e.g., [9–11]).

One of the most influential theories on speciation, Jürgen Haffer’s ‘refuge theory’ [12], which was widely followed in the 1980s and 1990s, postulates that elevated levels of species diversity in tropical rainforests are a product of rainforest contraction and savannah expansion during planetary cycles. However, earth-historic evidence for lowland rainforest contractions during glacial periods has failed to materialize (e.g. [13]), and tropical zoologists are amassing ever more evidence in support of species ages in most tropical lowland rainforest vertebrates that would pre-date Quaternary global glaciations [13–18]. Meanwhile, the role of ice ages in the diversification of tropical montane fauna has received relatively less attention, with most recent speciation events mainly attributed to tectonic processes such as mountain build-up (e.g. [19–23]).

The Andes are arguably the most species-rich area on Earth, with levels of taxon diversity along their eastern rim considerably exceeding most other regions in the world for many animal classes [24, 25]. While the southern Andes in Chile, Peru, Argentina and Bolivia are high enough for limited glaciation, a large part of the Andes would have been unaffected by the advance of ice sheets during periods of global cooling. However, global ice ages may have substantially affected the biota of the Andes through global drops in temperature by up to 8 °C [26], which would have led to a downslope shift of various assemblages of montane forest habitat possibly by several hundred meters [27, 28]. Many montane species that are nowadays restricted to fragmented pockets of mountaintop habitat isolated from neighboring populations by valleys may have enjoyed increased connectivity during glacial periods when their montane forest would have linked up across lower-lying ridges. There is limited research that has addressed the role of Pleistocene climate change in potentially counteracting speciation by increasing connectivity among isolated populations, especially in such hyper-diverse areas as the Andes that are known to be dominated by species of young age [21, 29].

In this study, we investigate patterns of gene flow in a young Andean-centered radiation of tyrant-flycatchers, the so-called ‘montane *Elaenia* clade’ [20]. In particular, we are interested in one species assemblage within the montane *Elaenia* clade, the so-called ‘*albiceps* complex’, that consists of three forms seemingly closely related to one another [30] but with differing levels of phenotypic and genetic divergence. Using a variety of population genetic and phylogenetic approaches, including coalescent simulations, and ~5.5 kb of DNA sequence data from dozens of samples from across the entire range of these species, we test whether recent gene flow among members of the ‘*albiceps* complex’, if any, is uniform across time or occurred disproportionately during the most recent period of global cooling. We also analyze whether patterns of gene flow support the hypothesis of character displacement having led to pronounced differences in phenotypic characters between two sympatric members of this complex, compared to less phenotypic differentiation in comparisons involving non-sympatric forms. Our study is one of few rigorous tests of the impacts of Pleistocene climate change on Neotropical montane biota that is based on a combination of acoustic, morphological and genetic data.

## Methods

### Bioacoustic analysis

We compiled a total of 50 vocal recordings of *albiceps*, *chilensis* and *pallatangae* from public sound libraries, including xeno-canto (http://www.xeno-canto.org) and Macaulay Library (http://macaulaylibrary.org). Details of all recordings analyzed, including localities, dates, and names of recordists, are provided in Additional file 1: Table S1. All our 50 recordings are homologous with one another, constituting a monosyllabic call that is the most commonly-heard vocalization given by montane *Elaenia* species, not the more complicated polysyllabic ‘dawn song’ [31]. This monosyllabic call note is distinctly different among the three *Elaenia* forms compared, but even so, we detected a limited number of obvious misidentifications that had been lodged with the sound libraries. We excluded recordings that may possibly be misidentified and ensured that the geographic locality of the recordings included in the analyses is in agreement with the newly assigned taxon’s distribution in every case. Using RAVEN PRO Version 1.5 (Bioacoustics Research Program, Cornell Laboratory of Ornithology, Ithaca, NY, USA), we measured seven vocal parameters: (i) call duration, (ii) proportion of time to reach maximum frequency, (iii) minimum frequency, (iv) maximum frequency, (v) peak frequency (defined as the frequency with the highest amplitude), (vi) ratio of bandwidth at maximum frequency versus at the start of the call, and (vii) ratio of bandwidth at maximum frequency versus at the end of the call (Additional file 1: Table S2). We used default settings of RAVEN PRO, except for window size, which was adjusted to 1024 to show optimal resolution of the spectrograms across all three taxa. We performed principal component analysis (PCA) on the vocal dataset to assess bioacoustic differences across the three taxa in R 3.2.1 [32].

To further highlight the difference in vocal traits, we calculated pairwise Euclidean distances between samples of different pairs of groups (*albiceps-chilensis*, *chilensis-pallatangae* and *albiceps-pallatangae*) by using their PCA coordinates from principal component 1 (PC1) and principal component 2 (PC2), as the latter two accounted for most of the variation within the dataset. We then used R to perform a Tukey’s test (as distances were normally distributed) to assess if these pairwise between-group distances are significantly different from one another.

### DNA sampling, extraction and sequencing

**Fig. 1:**
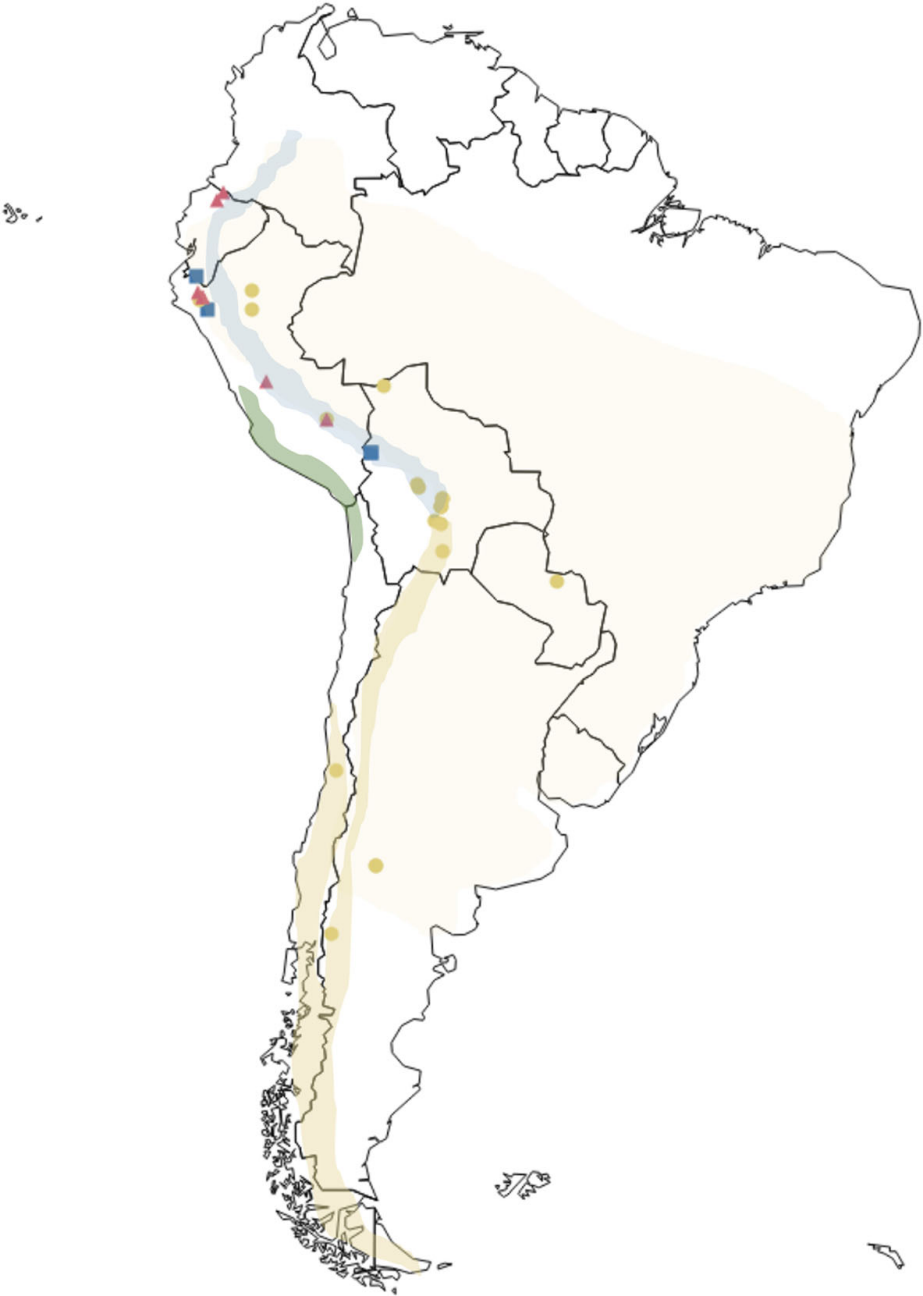
Map depicting the sampling and distribution of the ‘*albiceps* complex’. Dark mustard color depicts breeding range and light mustard depicts wintering range of the taxon *chilensis*. Blue color depicts all-year distribution of sympatric taxa *albiceps* and *pallatangae*. Green depicts the all-year distribution of the taxon *modesta*, which is unsampled in this study. Sampling localities for each taxon are depicted using symbols: *albiceps* in blue squares, *chilensis* in mustard circles and *pallatangae* in pink triangles. Map adapted from Fitzpatrick et al. [79] with minor changes

We used DNA extracts of samples that had been sourced for previous studies by Rheindt et al. [20, 30]. We sequenced DNA for 46 tissue samples across nine *Elaenia* species from various museums (Fig. 1; Additional file 1: Table S3), including all species belonging to a monophyletic clade of *Elaenia* flycatchers of primarily montane distribution, termed the ‘montane *Elaenia* clade’ by Rheindt et al. [21, 29], as well as a sample of *E. chiriquensis* (the sister taxon of the ‘montane *Elaenia* clade’) as an outgroup. Taxonomic treatment follows the recommendations by Rheindt et al. [20, 30].

In this study we sequenced nine loci (eight nuclear and one mitochondrial). Five of these (q4, q6, q8, q25, q26) are from among a panel of 26 anonymous loci distributed throughout the genome, primers for which were randomly generated using established protocols [33] and trialed for this project (Additional file 1: Table S4). These five loci yielded clear PCR products and were hence chosen over the remaining anonymous loci. One of our nine loci (02401) was chosen because it amplified best among a panel of 11 loci selected from a list of 242 anonymous markers proposed by Backström et al. [34] for population-genetic studies in birds. Another three loci, mitochondrial NADH dehydrogenase subunit 2 (ND2), β-fibrinogen intron 5 (Fib5) and tyrosinase related protein 1 (Tyrp1), are more conventional population-genetic and phylogenetic markers, two of which have amplified *Elaenia* DNA well in the past (ND2, Fib5; [20, 30]). Recent genomic data reveal that Tyrp1 is sex linked (present in the Z chromosome) in birds so sequence information of this locus was analyzed accordingly [35]. General PCR conditions largely followed Rheindt et al. [20], with primers and annealing temperatures for the nine loci provided in Additional file 1: Table S4. We manually edited all sequence chromatograms following Rheindt et al. [30]. Heterozygous sites were scored as degenerate sites based on established conventions of the International Union of Pure and Applied Chemistry (IUPAC), and were retained as one sequence per individual. All sequences generated in this study are available on GenBank (KY448474 - KY448797). We calculated the number of variable sites and parsimony-informative sites for our dataset in MEGA6 [36].

### Tests for intragenic recombination and neutrality

We performed genetic analysis for recombinant detection (GARD) [37] in order to assess the presence of intragenic recombination for the nuclear loci within our dataset. We conducted separate GARD analyses for each locus in the Datamonkey webserver [38]. We also performed the Hudson-Kreitman-Aguadé (HKA) test [39] as implemented in the program HKA https://bio.cst.temple.edu/~hey/software to assess deviations from neutrality for all loci in our dataset. We used the program SITES https://bio.cst.temple.edu/~hey/software [40] to generate input for the HKA test. The HKA test was carried out for the dataset consisting of the *Elaenia* taxa *chilensis*, *albiceps* and *pallatangae* only as this was our focal group for testing gene flow and isolation.

### Genetic distance

We calculated net between-taxa mean distances in MEGA using raw p-distances. We computed distances separately for the mitochondrial gene ND2 and for the all the autosomal nuclear loci, allowing for pairwise deletion of missing data. For the nuclear dataset, we computed genetic distances for each nuclear locus and averaged across all loci.

### Phylogenetic reconstruction

We reconstructed the phylogeny of the ‘montane *Elaenia* clade’ using both sequence concatenation as well as species tree methods [41]. We concatenated sequences of all nuclear loci using Sequence Matrix v1.7.8 [42] and employed RAxML GUI v1.5 [43] to reconstruct a maximum likelihood (ML) tree under the GTR + gamma model, performing 100 runs using a bootstrap algorithm with 1000 replicates. The resulting tree was visualized in FigTree v1.4.2 [44]. We performed species tree reconstructions using the maximum pseudo-likelihood coalescent method MP-EST [45] implemented in the STRAW [46] webserver, as well as a Bayesian method implemented in *BEAST v1.8.2 [47]. The anonymous locus q25 could not be sequenced for *E. fallax* and hence was removed from species tree analyses.

For the MP-EST analysis we first obtained individual gene trees with 1000 bootstraps for the eight loci using RAxML following the same parameters as for the concatenated analysis. We further rooted each gene tree with the *E. chiriquensis* outgroup using the ReRoot module in the STRAW webserver. The species tree was then estimated in MP-EST. In the Bayesian species tree reconstruction we considered each locus as a separate partition. For each locus, we determined the substitution model through jModelTest v 2.1.7 [48, 49] (Additional file 1: Table S5). We used a separate relaxed molecular clock model for each locus and estimated relative clock rates, applying a Yule speciation process with random starting gene trees under two independent runs of 100 million generations each and sampling every 100th generation. We checked parameter convergence of both runs in Tracer v 1.6 [50] and combined them in LogCombiner 1.8.2 with a 50% burnin while resampling once per 1000 generations. Subsequently we constructed a maximum clade credibility tree using median heights in TreeAnnotator 1.8.2 while allowing for a further 10% burnin using a posterior probability limit of 0.95. The final tree was visualized in FigTree. In order to explore reticulations and possible species tree topologies, we also used the combined trees obtained from LogCombiner 1.8.2 to generate a representation of topological uncertainty of species tree in DensiTree [51] with a 10% burnin.

We further explored the mitochondrial phylogeny by determining mitochondrial ND2 haplotypes and constructing a phylogenetic network with the program PopArt 1.7 [52] using the TCS method [53].

### Genetic clustering and admixture

We investigated gene flow among the three taxa of the ‘*albiceps* complex’ within the ‘montane *Elaenia* clade’ (i.e., *chilensis, albiceps, pallatangae*) with Bayesian clustering and admixture analyses in BAPS 5.4 [54–56]. We performed separate analyses on the alignment of the autosomal nuclear loci and the mitochondrial gene ND2. We first performed mixture analyses with ten iterations for a specified genetic cluster (K) and obtained estimates of ancestry coefficients of each individual. Based on our preliminary observations from phylogenetic analyses we performed mixture analyses for both K = 2 and K = 3. We further used the results from mixture analysis to perform population assignment and admixture analysis at both K = 2 and K = 3 using default settings. BAPS requires phased data for nuclear loci, hence we phased our data using PHASE v2.1 [57, 58] with default settings as implemented in DnaSP 5 [59].

### Modeling gene flow with coalescent simulations

To further investigate gene flow among the three taxa *chilensis, pallatangae* and *albiceps*, we performed coalescent simulations of various evolutionary models in fastsimcoal2 v 2.5.2.2.21 [60] and selected the best model using an Approximate Bayesian Computation (ABC) framework in the R package ABC [61]. Fastsimcoal simulates data following the Wright-Fisher model of evolution assuming neutrality of the genetic markers, no recombination within loci and free recombination between loci and random mating. ABC based approaches can efficiently differentiate between genetic patterns arising from gene flow versus shared ancestral polymorphism [62–66]. Coalescent simulations were only carried out on loci that showed no significant evidence of intragenic recombination and deviation from neutrality. We simulated a no-gene flow model and various gene flow models in a three-population framework and used alternative topological arrangements resulting from species tree analyses as well as leveraging hypothesized earth-historic events to construct evolutionary models reflecting these scenarios.

We also removed the sex-linked locus Tyrp1 for this analysis and from the remaining set of eight loci (seven nuclear autosomal and one mitochondrial) we simulated sequences of the same length as obtained from our sequencing and alignments. We allowed for complete recombination between loci but no intra-locus recombination. We performed 1,000,000 simulations for each model and calculated summary statistics from both the observed data as well as simulated datasets using arlsumstats v. 3.5.2 [67]. We performed separate simulations for the alignment of nuclear loci and the mitochondrial gene ND2. We set priors for population size (10 to 100,000) based on information obtained from Rheindt et al. [30]. Following the species tree topology and net interspecies mitochondrial distances observed in this study, and accounting for the uncertainty of divergence estimates between these lineages, we applied broad priors for divergence time parameters (divergence between *chilensis* and *pallatangae*: 10,000 to 2,000,000; divergence between *albiceps* and ancestor of *chilensis* and *pallatangae*: 1,000,000 to 7,000,000). We chose a broad prior for migration based on our understanding of species biology (probability of migration: 1e-8 to 1e-4 per generation), with all priors following a uniform distribution. We performed all simulations assuming a constant population size. In our first set of models we assumed various continuous, symmetric and low gene flow scenarios alongside a no-gene flow model. We plotted prior and posterior distributions and verified that the simulations had effectively sampled from the prior distributions for our parameters of interest. We always considered *chilensis* and *pallatangae* as sister clades with a much more recent coalescence than the timing of coalescence between *albiceps* and the ancestral population of *chilensis-pallatangae.* Whenever necessary, we further refined our models to test more complex scenarios of asymmetric gene flow as well as of isolation and migration patterns.

We chose summary statistics that can explain differentiation between populations and their shared variability (Additional file 1: Table S6). Before performing model selection, we quantified model misclassification using both hard (confusion matrix; see [61]) and soft classification (mean posterior probability) schemes as implemented in the ABC package [61]. We estimated the posterior probability of each model from a subset of simulations closest to the observed data (tolerance level) using multinomial logistic regression in the ABC package [61].

### Ancestral state reconstruction

We performed ancestral character state reconstruction of underparts coloration using BayesTraits v 2.0 [68]. We used a discrete two state coding (yellow versus white) for the terminal species nodes on the tree. The traits for each taxon are: *pallatangae* (yellow), *chilensis* (white), *albiceps* (white), *frantzii* (white), *olivina* (yellow), *fallax* (white), *cherriei* (white), *martinica* (white), *chiriquensis* (white). We used the multistate module in BayesTraits and performed a Bayesian MCMC run with 2,000,000 iterations, including a 100,000 iteration burnin, sampling every 500 generations, and estimated the ancestral state of each node in the final Bayesian species tree. We used an exponential distribution with a hyperprior as suggested by the program manual. We checked for parameter convergence using the coda package [69] in R.

## Results

### Bioacoustic variation within the ‘*albiceps* complex’

**Fig. 2:**
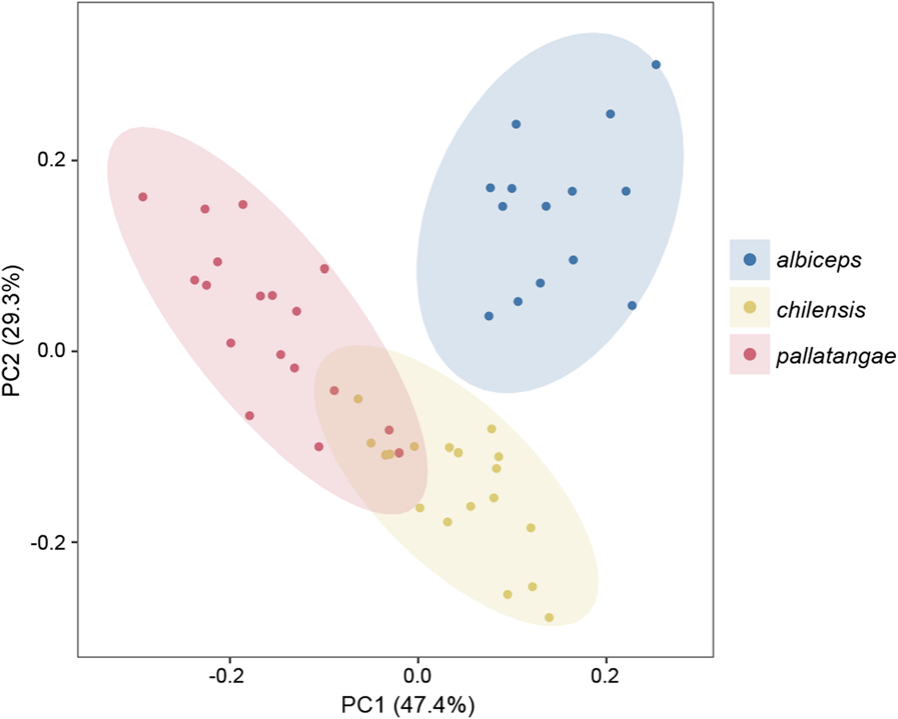
Principle component (PC) analysis of seven bioacoustic parameters revealing the acoustic variability within the ‘*albiceps* complex’. Variation explained by each axis is given in brackets. Ellipses represent 95% confidence intervals of the PC scores for each taxon

In general, the main call notes of *albiceps*, *chilensis* and *pallatangae* consist of simple monosyllabic vocal elements. The call of *albiceps* is rough and burred, whereas it is more melodious and mellow in the other two taxa. PCA of vocal parameters of the three *Elaenia* taxa revealed *albiceps* is vocally distinct from *pallatangae* and *chilensis* (Fig. 2), with the main differences mostly a result of parameters that quantify timbre and call quality (Additional file 2: Figure S1)*.* On the other hand, ample bioacoustic overlap was present between the vocal traits of *pallatangae* and *chilensis* (Fig. 2), with a roughly clinal arrangement along a north-south axis mainly based on average frequency parameters (Additional file 2: Figure S1). The 95% confidence ellipse of *albiceps* showed no overlap with that of either *pallatangae* or *chilensis*, although it was spatially much closer to *chilensis* (Fig. 2). Tukey’s test on the Euclidean PCA distances between samples (Fig. 2) confirmed this result, indicating that the distance between *albiceps* and *pallatangae* was significantly greater than between *albiceps* and *chilensis* or between *chilensis* and *pallatangae* (*p* value <0.001).

### DNA sequencing

We obtained 5573 bp of sequence data from across seven autosomal nuclear loci (3711 bp), one Z-linked locus (Tyrp1, 744 bp) and one mitochondrial locus (ND2, 1118 bp). Although we did not manage to sequence all 46 samples for all loci, we generated sequences for 67.4% of all sample–locus combinations (Additional file 1: Table S7). The number of variable sites varied between 13 and 246 per locus and the observed number of parsimony-informative sites per locus varied between 7 and 176 (Additional file 1: Table S8).

### Recombination and neutrality

None of the loci showed any significant evidence of recombination. We performed pairwise HKA test comparisons and did not observe significant deviation from neutrality in any locus (all pairwise *P* values >0.01; Additional file 1: Table S9). Thus we proceeded to carry out coalescent simulations on all eight loci (total 4829 bp).

### Phylogenetic reconstruction

**Fig. 3:**
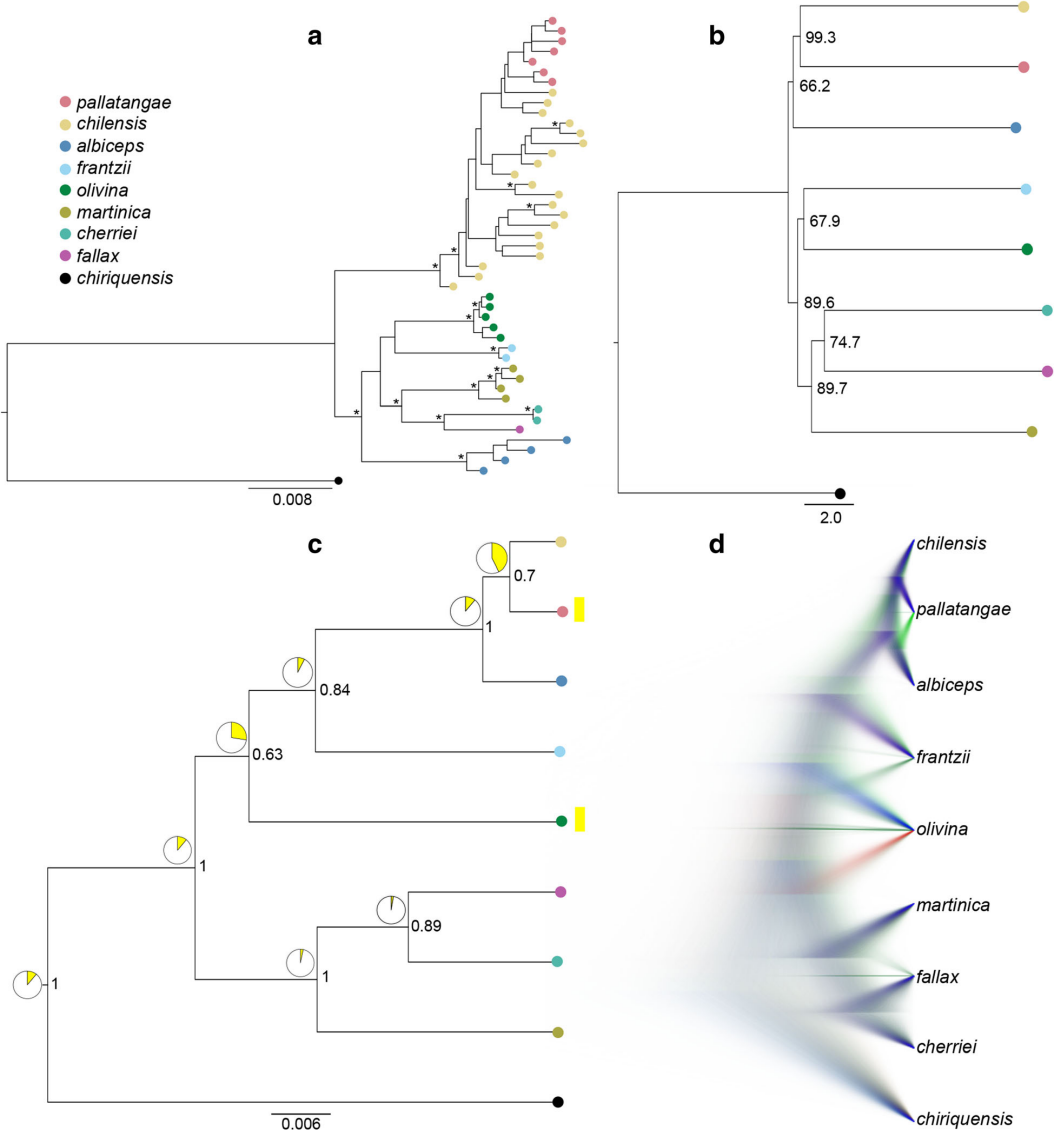
Phylogenetic reconstructions of the montane *Elaenia* clade: **a** Concatenation method (RAxML); nodes recovered with a bootstrap support of >70% are marked with an asterisk. **b** Maximum pseudo-likelihood estimation of species tree (MP-EST); node values denote bootstrap support. **c** Maximum clade credibility species tree (*BEAST) showing posterior nodal support values and probability of white or yellow underparts depicted using pie charts; taxa with yellow underparts are highlighted using yellow bars on the right. **d** Topological uncertainty of species tree (*BEAST) shown graphically in DensiTree to identify possible reticulations. Individual samples are color-coded based on species identity in panels **a**, **b** and **c**

In agreement with previous research using fewer loci [30], we observed that *chilensis* and *pallatangae* form a monophyletic complex using both ML concatenation and the two species tree methods (Fig. 3), albeit with variable support across species tree methods (Fig. 3b and c). However, beyond this arrangement, the ML concatenated tree and the species trees recovered somewhat incongruent topologies. The concatenated tree topology is in broad agreement with the phylogeny recovered by Rheindt et al. [30] in placing *albiceps* away from the *chilensis-pallatangae* cluster and with the remaining members of the montane *Elaenia* clade (Fig. 3a). Species tree analyses either produced low branch support regarding the placement of *albiceps* (Fig. 3b) or revealed significant posterior support for a monophyletic clade comprising *albiceps*, *chilensis* and *pallatangae* (Fig. 3c), henceforth called the ‘*albiceps* complex’. We also observed a high level of possible reticulations and discordances among gene trees, and a Densitree visualization of the Bayesian species tree suggests extensive reticulation within the ‘*albiceps* complex’ (Fig. 3d).

**Fig. 4:**
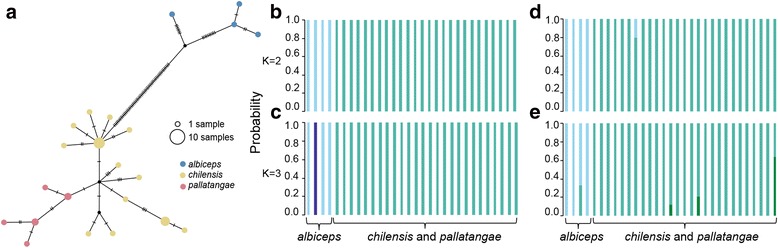
Phylogenetic network (PopArt) and population genetic assignment (BAPS) of the ‘*albiceps* complex’. **a** Mitochondrial ND2 network. **b** and **c** Bayesian clustering of individuals based on mitochondrial ND2 for K = 2 and K = 3. **d** and **e** Bayesian clustering of individuals based on seven autosomal loci for K = 2 and K = 3

The haplotype network revealed the presence of two major groups, one represented by *chilensis* and *pallatangae*, and the other represented by *albiceps* (Fig. 4a). All *pallatangae* haplotypes formed a distinct subgroup nested within *chilensis*, suggestive of a recent origin from within the *chilensis* cluster with potential recent isolation and a possible lack of recent mitochondrial gene flow. In contrast, *chilensis* haplotypes resemble a star shaped network suggestive of potential past expansion episodes (Fig. 4a).

Genetic distance estimates within the ‘montane *Elaenia* clade’ revealed that *chilensis* and *pallatangae* are genetically very close to each other both in nuclear as well as mitochondrial sequence data (Table 1) and together exhibit a divergence from *albiceps* in the mitochondrial gene ND2 that is typically considered a species-level divergence (for comparative mitochondrial divergences within *Elaenia*, see Rheindt et al. [20]). Similarly, we also observed that both *chilensis* and *pallatangae* are almost equidistant from *albiceps* (Table 1).

**Table 1:** Raw mean pairwise p-distances for all nuclear (nDNA; below diagonal) and mitochondrial (mtDNA; above diagonal) DNA sequences

nDNA/mtDNA	*albiceps*	*chilensis*	*pallatangae*	*fallax*	*cherriei*	*frantzii*	*martinica*	*olivina*	*chiriquensis*
*albiceps*	*	0.072	0.075	0.052	0.055	0.04	0.052	0.049	0.083
*chilensis*	0.003	*	0.002	0.078	0.072	0.068	0.072	0.066	0.087
*pallatangae*	0.003	0.001	*	0.078	0.073	0.071	0.07	0.068	0.087
*fallax*	0.012	0.012	0.013	*	0.052	0.057	0.052	0.055	0.089
*cherriei*	0.011	0.01	0.011	0.003	*	0.058	0.058	0.053	0.082
*frantzii*	0.009	0.012	0.009	0.016	0.015	*	0.056	0.047	0.081
*martinica*	0.007	0.008	0.008	0.007	0.006	0.01	*	0.056	0.081
*olivina*	0.008	0.009	0.009	0.011	0.009	0.009	0.006	*	0.078
*chiriquensis*	0.008	0.006	0.008	0.012	0.012	0.016	0.010	0.010	*

### Bayesian clustering and gene flow

We truncated missing data at both terminal sequence ends and used the resulting alignment of 2802 bp of nuclear data for phasing to perform BAPS analysis. However, we did not use the phased alignment for the aforementioned phylogenetic reconstructions because the 909 bp loss in alignment length led to lower resolution. Clustering analyses of both nuclear and mitochondrial markers across the three members of the ‘*albiceps* complex’ revealed the presence of two major genetic clusters, both at K = 2 and K = 3, with *chilensis* and *pallatangae* belonging to a single cluster (Fig. 4b-e). Increasing cluster numbers from K = 2 to K = 3 did not add further obvious taxonomic or geographic resolution among members of the ‘*albiceps* complex’ (Fig. 4b-e).

### Modeling gene flow

#### Nuclear loci

**Fig. 5:**
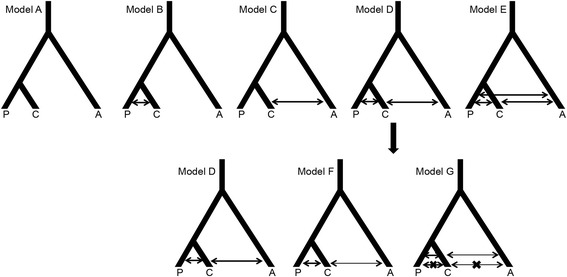
Gene flow models simulated for Approximate Bayesian computation. Abbreviation: A- *albiceps*, C- *chilensis* and P- *pallatangae*. See Tables 2 and 3 for a detailed description of models

An initial set of ABC analyses of five models (Fig. 5; Tables 2 and 3; models A-E) revealed that the no gene flow model (model A) was a poor fit to our data (tolerance level 10%), ruling out shared ancestral polymorphisms between *chilensis* – *pallatangae* and *chilensis* – *albiceps* as the sole explanation for the observed evolutionary history of the three *Elaenia* taxa under study (Figs. 3 and 4). Instead a continuous gene flow model between *chilensis* – *pallatangae* and *chilensis* – *albiceps* (model D) was best supported, returning the highest posterior probability (Table 2) and a higher Bayes factor than the next most well-supported model, which assumes migration among all three lineages (model E; Additional file 1: Table S10A). However, as model D is nested within the more complex model E (all migration model, Fig. 5; Tables 2 and 3;), the two were not greatly differentiated based on both hard and soft classifications (Additional file 1: Tables S11A and S12A). Goodness of fit test for model D suggested that the observed summary statistics fall within the prior distribution (*p* value = 0.059). Given the high posterior probability and high Bayes factor of model D (Fig. 5; Table 2; Additional file 1: Table S10A), we further used this model to determine if our genetic data can differentiate between an equal gene flow model (model D itself, Fig. 5; Tables 2 and 3), an asymmetrical gene flow model (model F, Fig. 5; Tables 2 and 3) and an isolation-migration model (model G, Fig. 5; Tables 2 and 3). The isolation-migration model (model G) assumes no gene flow during the current interglacial period of globally increased temperatures (roughly after 20 KYA in the Andes [70, 71]) but continuous gene flow during the period of global cooling prior to that. The rationale behind inferring a lack of gene flow during warmer, interglacial times as opposed to colder, glacial periods is based on the shift of montane habitat to lower elevations during cooler periods, allowing high-elevation biota such as the ‘montane *Elaenia* clade’ to engage in gene flow among different mountain ridges. Model selection at 5% tolerance suggested that this isolation-migration model (model G, Fig. 5) fits the data better than the continuous gene flow models (models E and F, Fig. 5; Table 3; Additional file 1: Table S10B) and could be well differentiated from other nested competing models (Additional file 1: Tables S11B and S12B). While the goodness of fit for model G was poor (*p* value = 0.037), posterior predictive checks revealed that all our observed summary statistics emerged within the prior distribution, suggesting that the model was suitable for parameter estimation (data not shown). We further performed parameter estimation for the isolation-migration model (model G, Fig. 5) by implementing the neuralnet algorithm at a 1% tolerance level (Table 4), demonstrating that all three lineages harbor a moderate effective population size (median between 70,000 and 85,000). Additional parameter estimates suggested that *chilensis* and *pallatangae* split on the order of a few hundreds of thousands of generations ago and divergence between the ancestral population of these two lineages and *albiceps* occurred roughly a few million generations ago (see Table 4 for exact median, mode, minimum and maximum estimates). We observed low levels of gene flow, with an approximate probability of migration of 1 in 10,000 between *chilensis* and *pallatange* each generation and 1 in 25,000 between *chilensis* and *albiceps* in each generation (median estimates; Table 4).

**Table 2:** Posterior probabilities for all models based on multinomial logistic regression; Models tested using both nuclear loci and mitochondrial ND2

Model set 1	Model code	Nuclear data	mtDNA data
No gene flow	Model A	0.0	0.0559
Gene flow between *chilensis* and *pallatangae* only	Model B	0.0	**0.8749**
Gene flow between *chilensis* and *albiceps* only	Model C	0.0018	0.0008
Gene flow between both *chilensis* and *pallatangae*, and *chilensis* and *albiceps*	Model D	**0.6647**	0.0598
Gene flow among all three lineages	Model E	0.3335	0.0086

Model codes refer to Fig. 4. Posterior probability of the best model is indicated in bold font

Abbreviation: *mtDNA* mitochondrial ND2

**Table 3:** Posterior probabilities for all models based on multinomial logistic regression; models specifically tested for nuclear loci only

Model set 2	Model code	Nuclear data
Equal gene flow between both *chilensis* and *pallatangae*, and *chilensis* and *albiceps*	Model D	0.2005
Higher rate of gene flow between *chilensis* and *pallatangae* compared to *chilensis* and *albiceps* (asymmetric gene flow model)	Model F	0.0733
Asymmetric gene flow model with gene flow until the last 20,000 years and isolation thereafter (isolation-migration model).	Model G	**0.7262**

Model codes refer to Fig. 4. Posterior probability of the best model is indicated in bold font

**Table 4:** Parameter estimation for the best supported model using; nuclear loci (model G, refer Fig. 5 and Tables 2 and 3 for more details)

	N_e_ *albiceps* (number of individuals)	N_e_ *chilensis* (number of individuals)	N_e_ *pallatangae* (number of individuals)	Time of divergence between *chilensis* and *pallatangae* (number of generations)	Time of divergence between *chilensis pallatangae* clade and *albiceps* (number of generations)	Migration rate between *chilensis* and *pallatangae* (probability of migration per generation)	Migration rate between *chilensis* and *albiceps* (probability of migration per generation)
Minimum	1.24E + 04	2.41E + 04	4.24E + 02	5.56E + 04	1.11E + 06	2.08E-06	8.98E-07
Weighted 2.5%	3.12E + 04	4.92E + 04	3.11E + 04	1.62E + 05	2.62E + 06	2.16E-05	4.64E-06
**Weighted Median**	**7.66E + 04**	**8.50E + 04**	**7.38E + 04**	**6.69E + 05**	**5.78E + 06**	**7.47E-05**	**2.63E-05**
Weighted Mean	7.35E + 04	8.20E + 04	7.14E + 04	7.11E + 05	5.52E + 06	6.99E-05	3.14E-05
Weighted Mode	9.47E + 04	9.50E + 04	7.97E + 04	4.68E + 05	6.67E + 06	9.36E-05	1.35E-05
Weighted 97.5%	9.91E + 04	9.93E + 04	9.82E + 04	1.51E + 06	6.95E + 06	9.89E-05	7.95E-05
Maximum	1.00E + 05	1.00E + 05	1.00E + 05	1.98E + 06	7.00E + 06	9.99E-05	9.89E-05

Weighted Median values are highlighted in bold

#### Mitochondrial gene ND2

We carried out gene flow modeling for the mitochondrial locus ND2 separately, and found that a model encompassing low migration between *chilensis* and *pallatangae* and a lack of migration between *albiceps* and the latter two lineages was best supported by the data (model B, Fig. 5; Additional file 1: Tables S10C and S12C). This model shows the maximum posterior probability (Table 2), a significantly higher Bayes factor compared with all other models (Additional file 1: Table S10C), and could be effectively differentiated from all other models in the confusion matrix (Additional file 1: Table S11C). The poor goodness of fit of this model (*p* = 0) was probably caused by an excess of the observed number of segregating sites and mean number of pairwise differences within *albiceps* as indicated by our posterior predictive checks (data not shown). A comparably high number of segregating sites within *albiceps* suggests either population subdivision or population expansion. However, as the sample size of *albiceps* was lower than for the other two taxa, we could not further increase the quality of our models by incorporating subdivision or demographic expansion. Parameter estimation for ND2 suggests a mitochondrial effective population size of around 20,000 for each of the three lineages (Table 5). The time of divergence between *chilensis* and *pallatangae* is estimated at ~900,000 generations before present, whereas separation between the ancestral population of these two lineages and *albiceps* occurred ~2.5 million generations ago (Table 5), both estimates being within the same order of magnitude as the estimates derived from the nuclear loci (Table 4). However, the poor goodness of fit of the best mitochondrial model and the fact that the ND2 dataset only represents one linkage group remain a serious limitation.

**Table 5:** Parameter estimation for the best supported model using; mitochondrial ND2 (model B, refer Fig. 5 and Table 2 for more details)

	N_e_ *albiceps* (number of individuals)	N_e_ *chilensis* (number of individuals)	N_e_ *pallatangae* (number of individuals)	Time of divergence between *chilensis* and *pallatangae* (number of generations)	Time of divergence between *chilensis pallatangae* clade and *albiceps* (number of generations)	Migration rate between *chilensis* and *pallatangae* (probability of migration per generation)
Minimum	6.83E + 01	3.97E + 01	1.12E + 01	1.11E + 04	1.00E + 06	5.72E-08
Weighted 2.5%	3.57E + 03	4.80E + 03	3.67E + 03	1.24E + 05	1.10E + 06	2.52E-06
**Weighted Median**	**1.90E + 04**	**1.91E + 04**	**1.86E + 04**	**9.32E + 05**	**2.55E + 06**	**4.18E-05**
Weighted Mean	1.75E + 04	1.79E + 04	1.72E + 04	9.65E + 05	2.99E + 06	4.51E-05
Weighted Mode	2.31E + 04	2.34E + 04	2.33E + 04	7.31E + 05	1.77E + 06	8.11E-06
Weighted 97.5%	2.47E + 04	2.48E + 04	2.47E + 04	1.93E + 06	6.56E + 06	9.79E-05
Maximum	2.50E + 04	2.50E + 04	2.50E + 04	2.00E + 06	6.99E + 06	1.00E-04

Weighted Median values are highlighted in bold

### Reconstruction of ancestral character states

The character state reconstruction analysis revealed that white underparts are a more probable ancestral character of the ‘*albiceps* complex’ (Fig. 3c). In general, for all internal nodes within the ‘montane *Elaenia* clade’, white underparts are more probable ancestral state than yellow underparts (Fig. 3c).

## Discussion

Recurrent Quaternary episodes of global cooling, the so-called “ice ages”, are known to have had an immense impact on differentiation processes at higher latitudes [4–8], yet very little is known about their effects on the biota of the tropics, including such centers of global biodiversity as the Andes. Using phylogenetic and population-genetic methods in conjunction with ~5.5 kb of sequence data across dozens of individuals of an Andean flycatcher lineage, coupled with morphological and bioacoustic data, our results suggest bouts of gene flow connecting some flycatcher lineages during peaks of global cooling when montane forest habitat would have extended to lower elevations and linked different mountain ridges [27, 28], while the current interglacial period of increased temperatures seems to be characterized by impeded gene flow between those lineages (Figs. 4 and 5; Tables 2 and 3).

### Incongruence between species tree reconstructions and concatenation-based methods

Our concatenation-based tree of the montane *Elaenia* clade using eight nuclear loci and one mitochondrial gene (Fig. 3a) was in broad agreement with an earlier phylogenetic reconstruction based on only two loci that had also been concatenated [30], revealing a tight-knit grouping consisting of the forms *pallatangae* and *chilensis*, which, in turn, were deeply differentiated from the remaining six species-level lineages of the montane *Elaenia* clade. In contrast to the concatenation-based tree (Fig. 3a), one of our two species tree reconstructions (the one based on *BEAST; Fig. 3c) provides strong support for a placement of *E. albiceps* as sister to the *pallatangae-chilensis* duo, while the other species tree method (based on MP-EST) recovers the same arrangement but with negligible support (Fig. 3b). In these species tree arrangements, the three forms *pallatangae*, *chilensis* and *albiceps* together form what has been called the ‘*albiceps* complex’, a traditional taxonomic treatment that has long associated these three forms as close relatives (e.g. [31]). It is difficult to identify the causes for the strong disagreement between concatenation and species tree methods in the placement of *E. albiceps*. On the one hand, inherent methodological differences in these two approaches are known to lead to a frequent incidence of conflict between their resulting topologies [41, 72–75]. On the other hand, both approaches have different susceptibilities to methodological biases in terms of number of loci, average locus length, phasing and other factors [41, 76, 77].

In complicated radiations such as the ‘*albiceps* complex’ with a potentially rich history of inter-species gene flow, the future use of genome-wide DNA data is called for to achieve a final resolution of evolutionary trajectories. At the same time, our analyses allow for the conclusion that *E. albiceps* is a strongly diverged species distinct from *E. pallatangae*, and that the latter should include *E. p. chilensis* as a subspecies.

### Phenotypic differentiation across the Andes in the face of gene flow

Species tree methods agreed with population-genetic approaches (e.g. mitochondrial network, BAPS) in aligning *chilensis* with *pallatangae* on a shallow node at near-zero mitochondrial differentiation (Figs. 3b, c and 4; Table 1). Contrary to Rheindt et al.’s [30] study of a single nuclear locus, our analysis of seven nuclear autosomal loci points to shallow levels of nuclear differentiation between *chilensis* and *pallatangae* (e.g. Table 1). In fact, our ABC analyses suggest considerable levels of recent gene flow between *chilensi*s and *pallatangae*, at least up to a few thousand years ago (Tables 4 and 5). The two forms have never been considered conspecific because of their diverging underparts coloration (yellow versus white) providing them with superficial plumage differences that appear comparatively pronounced within the genus *Elaenia*. However, plumage differences are known to be less important in the reproductive isolation of tyrant-flycatcher species (Tyrannidae), especially as compared to bioacoustic differences [78]. Our bioacoustic analysis showed that the calls of *chilensis* and *pallatangae* resemble each other more closely than either of them resembles the call note of *E. albiceps*, with clinal vocal trends in the *pallatangae-chilensis* cluster from north to south along the Andes (Fig. 2). Together, the genetic and phenotypic evidence leads us to propose that *pallatangae* and *chilensis* are two well-differentiated subspecies of a single biological species, *E. pallatangae*, connected by frequent gene flow and contiguity in vocal traits.

### Gene flow during periods of glacial maxima

Our approximate Bayesian computations suggest a complicated evolutionary history of the ‘*albiceps* complex’ with variable bouts of gene flow among constituent members (model G, Fig. 5; Tables 2, 3, 4 and 5). While the detection of extensive gene flow between *pallatangae* and *chilensis*, along with their similar vocalizations, leads us to call for their inclusion within one biological species, there is also a genetic signal for reciprocal monophyly and much less regular gene flow between the taxa *albiceps* and *chilensis* (model G, Fig. 5; Tables 2, 3, 4 and 5). These two taxa are possibly parapatric in their breeding distributions, with *albiceps* in the northern and central Andes and *chilensis* in the southern Andes, but gene flow is conceivable in central Bolivia where they probably meet [79].

Paleoclimatological studies specifically from the La Paz and Cochabamba in Bolivia reveal that ice sheets had expanded greatly during the last ice age, pushing the tree line down the slope by at least 1000 m from its present elevation [27, 80–82]. Hence this region and Andean areas beyond would have provided habitat connectivity for various *Elaenia* species during glacial periods, thereby facilitating gene flow.

We conducted computations to test whether gene flow between *chilensis* and the other two forms of *Elaenia* flycatcher happens roughly uniformly across time (model F, Fig. 5; Tables 2 and 3), or whether there is a pronounced signal for gene flow to occur during periods of global cooling (=‘ice ages’) when Andean habitats shift down the slope in response to a drop in global temperatures (model G, Fig. 5; Tables 2 and 3; [27, 80–82]). The Quaternary has gone through multiple ice ages [1, 2, 27] that may have allowed for increased gene flow among Andean biota, but testing comparative coalescent models for all these ice ages is not possible for a dataset of our size as it may lack power to account for such complex scenarios. Therefore, we concentrated on the most recent period of global cooling (climaxing roughly at 20,000 years ago) that is most immediately previous to the present interglacial period of warmer global temperatures. Specifically, we tested whether the present interglacial would have been characterized by a lack of gene flow compared to bouts of gene flow during the previous period of global cooling. Our tests confirm that bouts of gene flow would have been more intense prior to the present interglacial period of warmer global temperatures (Table 3), attesting to the importance of Pleistocene montane habitat shifts in the Andes for gene flow among high-elevation biota.

While glacial periods are known to have been engines of population fragmentation at the colder latitudes of the northern hemisphere [5], this study is one of the first to demonstrate that glaciations may have had an opposite effect on super-diverse Neotropical mountain ranges, providing montane habitat connections between populations on adjacent ridges that would otherwise be isolated from one another for millions of years. On the surface, this mechanism seems to counteract speciation by providing recurrent gene flow between incipient lineages, but it may actually be one of the underlying reasons for the unparalleled species diversity of the Andes: Montane forest bridges during ice ages may help maintain the exceptional species diversity of the Andes and other tropical mountain ranges, while ice ages would expose temperate mountain chains to the spread of less hospitable cool-adapted habitats, such as treeline scrub, tundra or ice sheets, that would lead to widespread extinction events of forest biota.

### Phenotypic character displacement

Our analyses uncover a complicated pattern of gene flow between the taxon *chilensis* and two other closely related forms of *Elaenia* flycatcher. While the white-bellied *chilensis* is connected with the yellow-bellied *pallatangae* by extensive gene flow and vocal similarities (Figs. 2 and 4; Tables 2, 3, 4 and 5; Additional file 1: Tables S10 and S12) – to the extent that we place them in the same biological species – there seems to be sporadic gene flow also between white-bellied *chilensis* and white-bellied *albiceps*. Vocal differences between *albiceps* and the other two forms are more pronounced (Fig. 2; Table 2, Additional file 1: Tables S10A and S12A). When one additionally considers the near-complete sympatry between *albiceps* and *pallatangae* (Fig. 1), it becomes clear that *albiceps* is an independent species, and rare instances of gene flow between it and any other *Elaenia* taxon must be treated as inter-specific introgression [83].

**Fig. 6:**
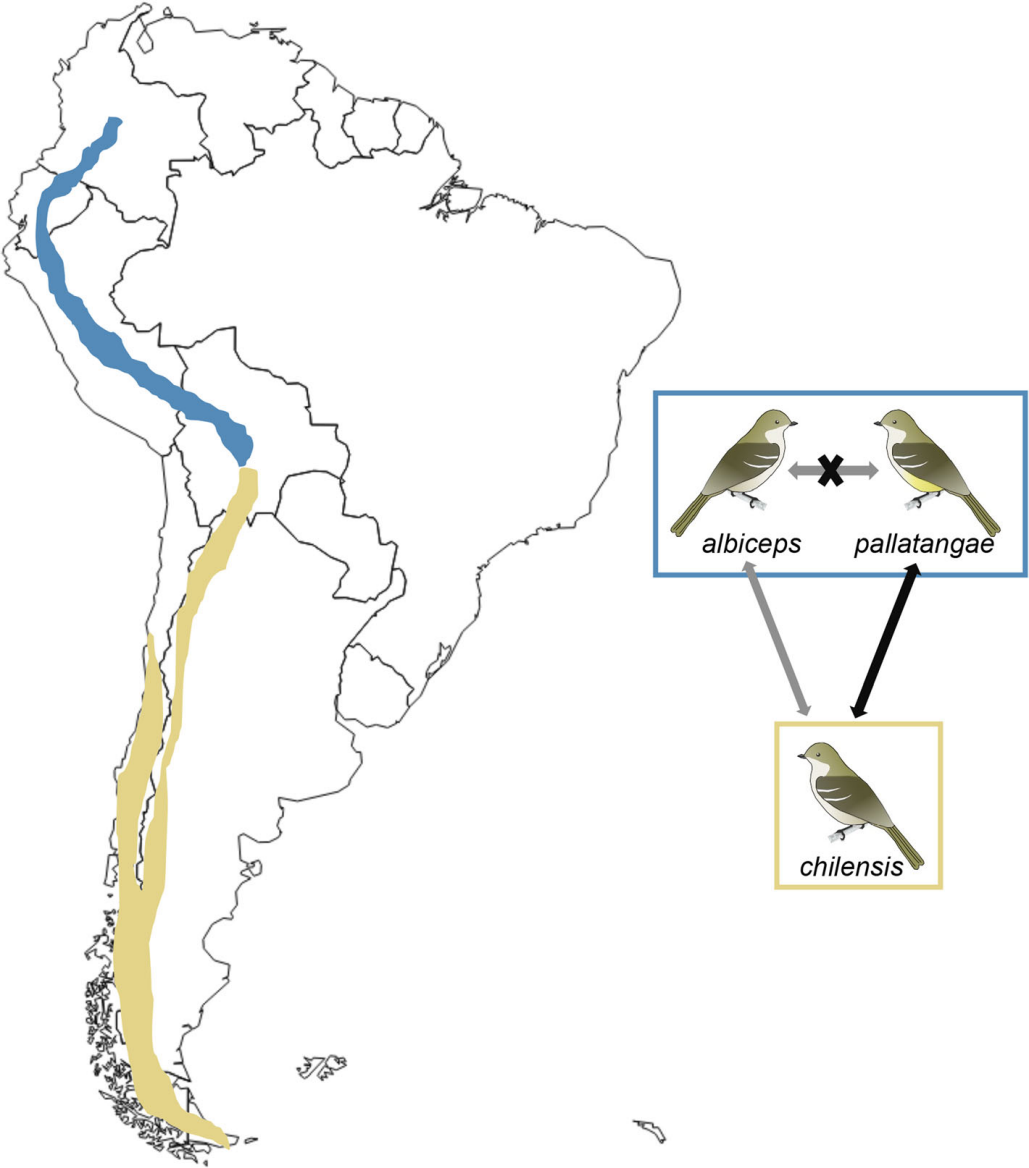
Figure illustrating the probable scenario of gene flow during the last glacial period and character displacement in phenotype within the ‘*albiceps* complex’. Mustard color depicts breeding range taxon *chilensis* and blue color depicts distribution of sympatric taxa *albiceps* and *pallatangae*. Diagrams of the birds depict the character displacement in belly color. Arrows depict gene flow, with black arrow depicting high gene flow and grey arrow depicting low gene flow. Crossed arrow depicts lack of gene flow

Our treatment of *chilensis* and *pallatangae* as members of one species, *E. pallatangae*, may appear to combine an unusual amount of plumage variation into a single species within such a morphologically conservative flycatcher genus. However, bioacoustic analysis (Fig. 2) confirms such a treatment, and indeed we argue that a process of character displacement may have contributed to the unusual variation of underparts coloration across *E. pallatangae* (including *chilensis*) (Fig. 6). In the northern and central Andes (Fig. 1), where sympatric with *E. albiceps*, populations of *E. pallatangae* have evolved a yellow underparts coloration (Figs. 3c and 6), possibly to set themselves apart from their closely related congeners and to avoid hybridization. In contrast, in the southern Andes where *E. albiceps* drops out in distribution, there has been no evolutionary pressure for the local form *E. p. chilensis* to relinquish its ancestral white underparts coloration (Figs. 3c and 6). Vocal evidence points to a similar pattern in which the burry call of *E. albiceps* differs from the more clear and melodious call of *E. pallatangae* (including *chilensis*) in general quality and timing (Fig. 2), but additionally differs from the higher-pitched call of sympatric populations of *E. pallatangae* in peak frequency and other frequency parameters whereas parapatric *E. p. chilensis* has been under no pressure to evolve a higher-pitched call, away from the equal-pitched vocalizations of *E. albiceps* (Additional file 2: Figure S1). In summary, our morphological, bioacoustic and genetic results point to a lack of detectable gene flow between the sympatric species *E. albiceps* and *E. p. pallatangae* in the face of heightened phenotypic (including bioacoustic) differentiation. In contrast, phenotypic (including bioacoustic) differences are much less pronounced between the largely non-overlapping *E. albiceps* and *E. p. chilensis*, and we detected discernible levels of gene flow between them occurring before the present interglacial period. Such a pattern is compatible with character displacement (Fig. 6).

## Conclusion

We studied the effects of Pleistocene climatic fluctuations on speciation dynamics in the ‘*albiceps* complex’ of Andean *Elaenia* tyrant-flycatchers. Using multi-locus sequence data as well as morphological and bioacoustics data we asked if ice ages have affected diversification within this group through habitat shifts in the montane biome. Our analyses reveal that periods of global cooling have bolstered gene flow among parapatric lineages. We also found strong evidence of reproductive isolation between sympatric lineages, and a pattern of character displacement in vocal and plumage traits from areas of sympatry to those of allopatry. Our findings provide a compelling mechanistic explanation for diversification and character displacement in natural populations within the Andean biodiversity hotspot.

## Additional files

Additional file 1: Supporting tables. (**Tables S1-** **S12**). (DOCX 71 kb)

Additional file 2: Figure S1. Loadings of the parameters used for principle component analysis of vocal characters (see Fig. 2 for details). Parameters are coded alphabetically. Parameter details are provided in Additional file 1: Table S2. (PDF 289 kb)
