# Supplementary material for: Gene flow during glacial habitat shifts facilitates character displacement in a Neotropical flycatcher radiation
Source: BMC Evol Biol. 2017 Sep 1;17:210. doi: 10.1186/s12862-017-1047-3 (PMC5580441; doi:10.1186/s12862-017-1047-3)
Supplement: Supplementary file 1 — Supporting tables. (Tables S1- S12). (DOCX 71 kb) [file 12862_2017_1047_MOESM1_ESM.docx]

Table S1: Details of sound recordings analyzed; abbreviations: XC – Xeno Canto sound library (www.xeno-canto.org); ML – Macaulay Library (www.macaulaylibrary.org).

| Taxon name | Recording identifier and source | Recording date | Locality | Coordinates | Recordist |
| --- | --- | --- | --- | --- | --- |
| *albiceps* | XC260860 | May-09 | Napo, Ecuador | -0.589, -77.877 | Mitch Lysinger |
| *albiceps* | XC258714 | Mar-99 | Celica, Ecuador | -4.001, -79.950 | John Moore |
| *albiceps* | XC222674 | 11-Jan-08 | Cajamarca, Peru | -7.170, -78.500 | Peter Boesman |
| *albiceps* | XC30082 | 15-Feb-09 | Cusco, Peru | -13.728, -71.618 | David Geale |
| *albiceps* | XC3030 | 20-Nov-97 | Tile, Chile | -18.667, -70.267 | Bennett Hennessey |
| *albiceps* | XC2043 | 5-Dec-93 | La Paz, Bolivia | -16.931, -67.176 | Sjoerd Mayer |
| *albiceps* | XC149110 | 13-Sep-13 | Santa Cruz dept., Bolivia | -17.820, -64.678 | Dan Lane |
| *albiceps* | XC30083 | 15-Feb-09 | Cusco, Peru | -13.728, -71.618 | David Geale |
| *albiceps* | XC120584 | 28-Mar-12 | Pichincha, Ecuador | 0.100, -78.350 | Lars Lachmann |
| *albiceps* | XC17225 | 17-Jan-08 | Cusco, Peru | -13.618, -71.717 | Israel Aragon |
| *albiceps* | XC296384 | 14-May-15 | La Paz, Bolivia | -15.827, -68.644 | Ross Gallardy |
| *albiceps* | ML173883 | 21-Sep-12 | Chupón, Peru | -13.246, -73.501 | Mark Robbins |
| *albiceps* | ML174019 | 6-Oct-12 | Cuzco, Peru | -13.376, -73.137 | Mark Robbins |
| *albiceps* | ML78935 | 24-Oct-92 | Loja, Ecuador | -4.000, -79.500 | Mark Robbins |
| *chilensis* | XC326092 | 8-May-13 | Santa Cruz Cabrália, Brazil | -16.289, -39.335 | Fernando Igor de Godoy |
| *chilensis* | XC325629 | 12-May-12 | Porto Seguro, Brazil | -16.453, -39.255 | Fernando Igor de Godoy |
| *chilensis* | XC325628 | 10-May-12 | Taquara, Brazil | -15.985, -39.376 | Fernando Igor de Godoy |
| *E. chilensis* | XC295734 | 28-Mar-12 | Monte Alegre, Brazil | -24.278, -50.531 | Fernando Igor de Godoy |
| *chilensis* | XC225270 | 11-Mar-15 | Lago Colbún, Chile | -35.694, -71.223 | Lance Benner |
| *chilensis* | XC186273 | 8-Jun-11 | Passo de Camaragibe, Brazil | -9.316, -35.442 | Fernando Igor de Godoy |
| *chilensis* | XC171032 | 25-Nov-13 | Cabo de Hornos, Chile | -54.950, -67.633 | Omar Barroso |
| *chilensis* | XC46420 | 7-Feb-04 | Parque Nacional Lanín, Argentina | -40.160, -71.358 | Bernabe Lopez-Lanus |
| *chilensis* | XC45431 | 24-Feb-10 | Las Mellizas Wetland, Chile | -37.331, -72.401 | Daniel González Amat |
| *chilensis* | XC19510 | 8-Feb-06 | El Volcán (Puerto Octay), Chile | -41.010, -72.750 | Fabrice Schmitt |
| *chilensis* | XC17125 | 19-Jan-08 | below Farellones, Chile | -30.348, -70.332 | Doug Knapp |
| *chilensis* | XC16004 | 9-Feb-84 | Lago Roca, Argentina | -50.500, -72.750 | Niels Krabbe |
| *chilensis* | XC53274 | 1992-12-00 | Chubut, Argentina | -42.168, -71.638 | Felix Vidoz |
| *chilensis* | XC29346 | 1-Feb-00 | Calilegua, Argentina | -23.683, -64.883 | Niels Krabbe |
| *chilensis* | XC116022 | 30-Dec-12 | Quinta Región, Chile | -32.991, -71.171 | Daniel González Amat |
| *chilensis* | ML135822 | 24-Jan-05 | Lago Espejo (Villa La Angostura), Argentina | -40.687, -71.695 | Juan Areta |
| *chilensis* | ML523294 | 29-Dec-07 | Mendozal, Argentina | -34.815, -68.492 | Juan Areta |
| *chilensis* | ML210363 | 20-Nov-12 | Salta, Argentina | -24.510, -65.336 | Juan Areta |
| *pallatangae* | XC275963 | 9-Sep-02 | Imbabura, Ecuador | 0.364, -78.485 | Jonas Nilsson |
| *pallatangae* | XC264673 | Mar-91 | Imbabura, Ecuador | 0.364, -78.485 | Paul Coopmans |
| *pallatangae* | XC250813 | 3-Mar-08 | Upper Yunguilla Valley, Ecuador | -3.232, -79.281 | Niels Krabbe |
| *pallatangae* | XC250295 | 13-Apr-91 | Loja, Ecuador | -4.083, -79.950 | Niels Krabbe |
| *pallatangae* | XC250120 | 15-Feb-91 | El Oro, Ecuador | -3.387, -79.574 | Niels Krabbe |
| *pallatangae* | XC250115 | 22-Nov-98 | Azuay: Cebadillas 8 km SSE Pucará, Ecuador | -3.281, -79.422 | Niels Krabbe |
| *pallatangae* | XC248321 | 13-Aug-97 | Carchi, Ecuador | 0.830, -78.051 | Niels Krabbe |
| *pallatangae* | XC222705 | 28-Dec-94 | Tablas Monte, Bolivia | -17.150, -65.900 | Peter Boesman |
| *pallatangae* | XC88575 | 4-Oct-11 | Wayqecha, Cusco, Peru | -13.175, -71.588 | David Geale |
| *pallatangae* | XC2935 | 5-Oct-97 | Corani, Bolivia | -17.167, -65.893 | Bennett Hennessey |
| *pallatangae* | XC283660 | 26-Sep-15 | Santa Barbara, Ecuador | 0.617, -77.583 | Roger Ahlman |
| *pallatangae* | XC275964 | 8-Feb-99 | Cajanuma, Ecuador | -4.118, -79.176 | Jonas Nilsson |
| *pallatangae* | XC222706 | 5-Jan-08 | Abra Patricia Road, Peru | -5.833, -77.767 | Peter Boesman |
| *pallatangae* | XC47620 | 9-Jul-83 | Cusco, Peru | -13.167, -72.550 | Niels Krabbe |
| *pallatangae* | XC2082 | 10-Dec-93 | La Paz, Bolivia | -16.861, -67.205 | Sjoerd Mayer |
| *pallatangae* | ML147713 | 22-Mar-12 | Upa, Peru | -6.003, -77.823 | Fernando Angulo |
| *pallatangae* | ML147706 | 21-Mar-12 | Upa, Peru | -6.003, -77.823 | Fernando Angulo |

Table S2: Definition of parameters used for analyzing sound recordings.

| Parameter | Code used in Figure S1 | Definition |
| --- | --- | --- |
| Call duration | A | Duration of time from the beginning to the end of a single call |
| Proportion of time to reach maximum frequency | B | Proportion of element duration until maximum frequency of a call is reached |
| Minimum frequency | C | Lowest pitch as reflected by the lowest-frequency region of the trace on the spectrogram |
| Maximum frequency | D | Highest pitch as reflected by the highest-frequency region of the trace on the spectrogram |
| Peak frequency | E | Frequency of a motif at which the amplitude of the sound is highest. This is reflected by the darkest region of the trace, and computed automatically by Raven. |
| Ratio of bandwidth at maximum frequency versus at the start of the call | F | Bandwidth refers to the difference between highest and lowest frequency of a specific region on the spectrogram; this parameter is the ratio of the bandwidth at maximum frequency over the bandwidth at the start of the call |
| Ratio of bandwidth at maximum frequency versus at the end of the call | G | The ratio of the bandwidth at maximum frequency over the bandwidth at the end of the call |

Table S3: List of *Elaenia* tissue samples used in this study along with their tissue numbers, housing institutions and collecting localities; institutional abbreviations: AMNH – American Museum of Natural History, New York; UZMK – Universitetets Zoologiske Museum *København (Copenhagen University Zoological Museum); FMNH – Field Museum of Natural History, Chicago; LSUMNS – Louisiana State University Museum of Natural Science, Baton Rouge, Louisiana; UKNHM – University of Kansas Natural History Museum, Lawrence, Kansas;* SNMNH – Smithsonian Institution National Museum of Natural History, Washington, D.C.

| **Tissue number** | **Taxon name** | **Institution** | **Collection locality** |
| --- | --- | --- | --- |
| CJV150 | *albiceps* | AMNH | BOLIVIA: La Paz, Bautista Savaedra |
| CJV152 | *albiceps* | AMNH | BOLIVIA: La Paz, Bautista Savaedra |
| KOB125231 | *albiceps* | UZMK | PERU: Cajamarca, Llama-Huambos |
| KOB125234 | *albiceps* | UZMK | ECUADOR: Loja, Utuana |
| 120830 | *chilensis* | UZMK | BOLIVIA: Chuquisaca, Palmarcito |
| 125229 | *chilensis* | UZMK | BOLIVIA: Cochabamba, Cerro Khenwa-Sandra |
| 126222 | *chilensis* | UZMK | BOLIVIA: Chuquisaca, Tarabuco |
| 126225 | *chilensis* | UZMK | BOLIVIA: Chuquisaca, Palmarcito 18.35S/64.52W |
| 126226 | *chilensis* | UZMK | BOLIVIA: Chuquisaca, Palmarcito 18.35S/64.52W |
| 126260 | *chilensis* | UZMK | BOLIVIA: Chuquisaca, Sopachuy |
| 126570 | *chilensis* | UZMK | BOLIVIA: Cochabamba, Tablas Montes |
| 144936 | *chilensis* | UZMK | BOLIVIA: Chuquisaca, Montes Chapeados |
| 430029 | *chilensis* | FMNH | PERU: Cusco |
| B179 | *chilensis* | LSUMNS | PERU: Piura, KM 34 on Olmos – Bagua Chica Highway |
| B42164 | *chilensis* | LSUMNS | PERU: Loreto, 7 km SW Jeberos |
| B5399 | *chilensis* | LSUMNS | PERU: San Martin, 20 km NE Tarapoto towards Yurimaguas |
| B9305 | *chilensis* | LSUMNS | BOLIVIA: Pando, Nicolas Suarez, 12 km by road S of Cobija, 8 km W on road to Mucden |
| CJV273 | *chilensis* | AMNH | BOLIVIA: Santa Cruz, Caballeros, Amboró NP |
| CJV313 | *chilensis* | AMNH | BOLIVIA: Santa Cruz, Caballeros, Amboró NP |
| KU64 | *chilensis* | UKNHM | PARAGUAY |
| PRS1136 | *chilensis* | AMNH | ARGENTINA: Río Negro, Bariloche |
| PRS1734 | *chilensis* | AMNH | ARGENTINA: Neuquén, Anelo, Sierra Auca Mahuida |
| PRS1814 | *chilensis* | AMNH | ARGENTINA: Neuquén, Anelo, Sierra Auca Mahuida |
| RTC399 | *chilensis* | AMNH | CHILE: Metropolitana, Chacabuco, 4 km SSW from peak of Cerro El Roble, 1660 m |
| 125239 | *pallatangae* | UZMK | ECUADOR: Carchi, below Laurel |
| 125242 | *pallatangae* | UZMK | ECUADOR: Imbabura, Apuela Rd, 2800m |
| 433534 | *pallatangae* | FMNH | PERU: Cusco |
| B31835 | *pallatangae* | LSUMNS | PERU: Cajamarca, Quebrada Lanchal, 8 km ESE Sallique |
| B32050 | *pallatangae* | LSUMNS | PERU: Cajamarca, Quebrada Lanchal, 8 km ESE Sallique |
| B398 | *pallatangae* | LSUMNS | PERU: Piura, Cruz Blanca, 33 km by road SW Huancabamba |
| B8155 | *pallatangae* | LSUMNS | PERU: Pasco, Playa Pampa, 8 km NW Cushi on trail to Chaglla |
| B15284 | *chiriquensis* | LSUMNS | BOLIVIA: Santa Cruz |
| 331075 | *fallax* | FMNH | JAMAICA |
| JAG2175 | *cherriei* | AMNH | DOMINICAN REPUBLIC: Independencia, Sierra de Neiba |
| KU6293 | *cherriei* | UKNHM | DOMINICAN REPUBLIC |
| B5469 | *frantzii* | SNMNH | PANAMA |
| KU4901 | *frantzii* | UKNHM | EL SALVADOR |
| B11342 | *martinica* | LSUMNS | PUERTO RICO: Cabo Rojo, Llanos Costa, 0.5km NNW of the mouth of Arroyo Cazul |
| B11343 | *martinica* | LSUMNS | PUERTO RICO: Cabo Rojo, Llanos Costa, 0.5km NNW of the mouth of Arroyo Cazul |
| B2116 | *martinica* | SNMNH | ST. VINCENT (Lesser Antilles) |
| NKK784 | *martinica* | AMNH | CAYMAN ISLANDS: Grand Cayman |
| GFB2764 | *olivina* | AMNH | VENEZUELA: Bolívar, Auyán Tepui, Camp I, 1700m |
| GFB2904 | *olivina* | AMNH | VENEZUELA: Bolívar, Cerro Guanay, 1400m |
| GFB2936 | *olivina* | AMNH | VENEZUELA: Amazonas, Cerro Yavi, 2100m |
| GFB2960 | *olivina* | AMNH | VENEZUELA: Amazonas, Cerro Yutaje, 1700m |
| PEP1987 | *olivina* | AMNH | VENEZUELA: Amazonas, Tamacuari |

Table S4: Primer information and annealing temperatures for each locus.

| **Locus** | **Forward primer** | **Reverse primer** | **Annealing Temperature** |
| --- | --- | --- | --- |
| ND2  sequenced in two overlapping fragments | Fragment 1: L5215 [83]; Fragment 2: FRND2.1 [20] | Fragment 1: H5578 [83]; Fragment 2: H6315 [84] | 57 °C |
| Fib5 | Fib5 [85]:  CGCCATACAGAGTATACTGTGACA | Fib6 [85]: GCCATCCTGGCGATTCTGAA | 57 °C |
| P02401 | GAGAACCTGGAGTTCCTCAG | TCAAGTGTGTCAGGTTAGGAG | 57 °C |
| q4 | CAGCTCCCAGGGTTGTACTG | AGGCTGAGAGGGCTTAAAGG | 57 °C |
| q6 | ATGTCCAACCTGGGACTGAG | TCAGATATCCCTGCCCTGTC | 57 °C |
| q8 | ACCCCTGGTTTACACTGCAC | TGTGGTTCTTTTGGGGGTAG | 57 °C |
| q25 | TTGGCTCAGAGCACAGAGAG | TCCTCACCGAGAAAATAGGG | 59.8 °C |
| q26 | AGCAACGCAATTACGTGAAG | TGATGATCCCTTCAAATAGCC | 54.5 °C |
| Tyrp1 | GAAATGTTTGTTACTGCACC | AGAAGAGGCTGATGCAAC | 55 °C and 56 °C |

Table S5: List of the most likely evolutionary models for each locus as assessed through the Akaike Information Criterion in jModelTest. For locus identities, see Methods. For model names, see [47, 48].

| Locus | Best model |
| --- | --- |
| 02401 | TPM1+I+G |
| Fib5 | TPM1uf+G |
| q25 | TIM2+G |
| q26 | GTR+G |
| q4 | HKY+G |
| q6 | TrN+G |
| q8 | HKY |
| Tyrp1 | TPM2uf |
| ND2 | TrN+I+G |

Table S6: List of summary statistics used for Approximate Bayesian Computation.

| Symbol | Summary statistics |
| --- | --- |
| S | Absolute number of segregating sites for each taxon |
| Mean_S | Mean absolute number of segregating sites across the three taxa |
| SD_S | Standard deviation of number of segregating sites over taxa |
| Tot_S | Total number of segregating sites across all taxa |
| Pi | Mean number of pairwise differences for each taxon |
| Mean_Pi | Mean number of pairwise differences averaged over all taxon comparisons |
| SD_pi | Standard deviation of the mean number of pairwise differences across taxa |
| F_ST_ | Pairwise F_ST_ |

Table S7: Loci sequenced for each sample. For locus abbreviations, see Methods section.

| Tissue number | Taxon name | ND2 | Fib5 | P02401 | q4 | q6 | q8 | q25 | q26 | Tyrp1 |
| --- | --- | --- | --- | --- | --- | --- | --- | --- | --- | --- |
| CJV150 | *albiceps* | Yes | No | Yes | Yes | Yes | Yes | Yes | Yes | Yes |
| CJV152 | *albiceps* | Yes | Yes | Yes | Yes | Yes | Yes | Yes | Yes | Yes |
| KOB125231 | *albiceps* | Yes | No | Yes | Yes | Yes | Yes | Yes | Yes | Yes |
| KOB125234 | *albiceps* | Yes | Yes | Yes | Yes | Yes | Yes | Yes | Yes | Yes |
| 120830 | *chilensis* | Yes | Yes | Yes | Yes | Yes | Yes | Yes | Yes | Yes |
| 125229 | *chilensis* | Yes | Yes | No | Yes | No | Yes | No | Yes | Yes |
| 126222 | *chilensis* | Yes | Yes | No | Yes | Yes | Yes | Yes | Yes | Yes |
| 126225 | *chilensis* | Yes | Yes | No | Yes | Yes | Yes | No | Yes | Yes |
| 126226 | *chilensis* | Yes | Yes | Yes | Yes | Yes | Yes | Yes | Yes | Yes |
| 126260 | *chilensis* | Yes | Yes | No | Yes | Yes | Yes | Yes | Yes | Yes |
| 126570 | *chilensis* | Yes | Yes | No | Yes | Yes | Yes | No | Yes | Yes |
| 144936 | *chilensis* | No | Yes | No | Yes | Yes | Yes | Yes | No | No |
| 430029 | *chilensis* | Yes | Yes | Yes | Yes | Yes | Yes | Yes | Yes | Yes |
| B179 | *chilensis* | Yes | Yes | Yes | Yes | No | Yes | Yes | Yes | Yes |
| B42164 | *chilensis* | Yes | Yes | Yes | Yes | Yes | Yes | Yes | Yes | Yes |
| B5399 | *chilensis* | Yes | Yes | Yes | Yes | Yes | No | Yes | Yes | Yes |
| B9305 | *chilensis* | Yes | Yes | Yes | Yes | Yes | Yes | Yes | Yes | Yes |
| CJV273 | *chilensis* | Yes | Yes | Yes | Yes | Yes | Yes | Yes | Yes | Yes |
| CJV313 | *chilensis* | Yes | Yes | Yes | Yes | Yes | Yes | Yes | Yes | Yes |
| KU64 | *chilensis* | Yes | Yes | Yes | Yes | Yes | Yes | Yes | Yes | Yes |
| PRS1136 | *chilensis* | Yes | Yes | Yes | Yes | Yes | Yes | Yes | Yes | Yes |
| PRS1734 | *chilensis* | Yes | Yes | Yes | Yes | Yes | Yes | Yes | Yes | Yes |
| PRS1814 | *chilensis* | Yes | Yes | Yes | Yes | Yes | Yes | Yes | Yes | Yes |
| RTC399 | *chilensis* | Yes | Yes | Yes | Yes | Yes | Yes | Yes | Yes | Yes |
| 125239 | *pallatangae* | Yes | Yes | Yes | Yes | Yes | Yes | Yes | Yes | Yes |
| 125242 | *pallatangae* | Yes | Yes | No | Yes | Yes | Yes | Yes | Yes | Yes |
| 433534 | *pallatangae* | Yes | Yes | Yes | Yes | No | Yes | Yes | Yes | Yes |
| B31835 | *pallatangae* | Yes | Yes | Yes | Yes | Yes | Yes | Yes | Yes | Yes |
| B32050 | *pallatangae* | Yes | No | Yes | Yes | Yes | Yes | Yes | Yes | Yes |
| B398 | *pallatangae* | Yes | Yes | Yes | Yes | Yes | Yes | Yes | Yes | Yes |
| B8155 | *pallatangae* | Yes | Yes | Yes | Yes | Yes | Yes | Yes | Yes | Yes |
| B15284 | *chiriquensis* | Yes | Yes | Yes | Yes | Yes | Yes | Yes | Yes | Yes |
| 331075 | *fallax* | Yes | Yes | Yes | Yes | Yes | Yes | - | Yes | Yes |
| JAG2175 | *fallax* | Yes | Yes | - | Yes | Yes | Yes | Yes | Yes | Yes |
| KU6293 | *fallax* | Yes | Yes | Yes | Yes | Yes | Yes | Yes | Yes | Yes |
| B5469 | *frantzii* | Yes | Yes | Yes | Yes | Yes | Yes | Yes | Yes | Yes |
| KU4901 | *frantzii* | Yes | Yes | Yes | Yes | Yes | Yes | Yes | Yes | Yes |
| B11342 | *martinica* | Yes | Yes | Yes | Yes | Yes | Yes | Yes | Yes | Yes |
| B11343 | *martinica* | Yes | Yes | Yes | Yes | Yes | Yes | Yes | Yes | Yes |
| B2116 | *martinica* | Yes | Yes | Yes | Yes | Yes | Yes | Yes | Yes | Yes |
| NKK784 | *martinica* | Yes | Yes | Yes | Yes | Yes | Yes | Yes | Yes | Yes |
| GFB2764 | *olivina* | Yes | Yes | Yes | Yes | Yes | Yes | Yes | Yes | Yes |
| GFB2904 | *olivina* | Yes | Yes | Yes | Yes | Yes | Yes | Yes | Yes | Yes |
| GFB2936 | *olivina* | Yes | Yes | Yes | Yes | Yes | Yes | Yes | Yes | Yes |
| GFB2960 | *olivina* | Yes | Yes | Yes | Yes | Yes | Yes | Yes | Yes | Yes |
| PEP1987 | *olivina* | Yes | Yes | Yes | Yes | Yes | Yes | Yes | Yes | Yes |

Table S8: Sequence length, number of variable sites and parsimony-informative sites for each locus.

| Locus | Fragment length | Number of variable sites | Number of parsimony-informative sites |
| --- | --- | --- | --- |
| 02401 | 624 | 83 | 38 |
| Fib5 | 602 | 200 | 51 |
| q25 | 417 | 13 | 7 |
| q26 | 511 | 52 | 30 |
| q4 | 541 | 26 | 10 |
| q6 | 502 | 41 | 28 |
| q8 | 514 | 17 | 7 |
| Tyrp1 | 744 | 51 | 27 |
| ND2 | 1118 | 246 | 176 |

Table S9: Results of test for significant deviation from neutrality.

| Comparison | p value |
| --- | --- |
| *albiceps* vs *chilensis* | 0.51 |
| *albiceps* vs *pallatangae* | 0.02 |
| *chilensis* vs *pallatangae* | 0.81 |

Table S10: Bayes factors across models. For model specifications, see Table 2 and Fig. 5. A) Nuclear loci for model set 1 (see Table 2A); B) nuclear loci for model set 2 (see Table 2B); C) mitochondrial ND2 for model set 1 (see Table 2A).

Table S10A

|  | Model A | Model B | Model C | Model D | Model E |
| --- | --- | --- | --- | --- | --- |
| Model A | 1 | 6.80E+04 | 0 | 0 | 0 |
| Model B | 0 | 1 | 0 | 0 | 0 |
| Model C | 3.53E+06 | 2.40E+11 | 1 | 2.80E-03 | 5.50E-03 |
| Model D | 1.28E+09 | 8.71E+13 | 3.62E+02 | 1 | 1.99 |
| Model E | 6.42E+08 | 4.37E+13 | 1.82E+02 | 5.02E-01 | 1 |

Table S10B

|  | Model D | Model F | Model G |
| --- | --- | --- | --- |
| Model D | 1 | 2.7331 | 0.276 |
| Model F | 0.3659 | 1 | 0.101 |
| Model G | 3.6226 | 9.9007 | 1 |

Table S10C

|  | Model A | Model B | Model C | Model D | Model E |
| --- | --- | --- | --- | --- | --- |
| Model A | 1 | 0.06 | 67.71 | 0.94 | 6.52 |
| Model B | 15.65 | 1 | 1059.28 | 14.63 | 101.97 |
| Model C | 0.02 | 0.0 | 1 | 0.01 | 0.10 |
| Model D | 1.07 | 0.07 | 73.4 | 1 | 6.97 |
| Model E | 0.15 | 0.0 | 10.39 | 0.14 | 1 |

Table S11: Confusion matrix (hard classification) across models. For model specifications, see Table 2 and Fig. 5. A) Nuclear loci for model set 1 (see Table 2A); B) nuclear loci for model set 2 (see Table 2B); C) mitochondrial ND2 for model set 1 (see Table 2A).

Table S11A

|  | Model A | Model B | Model C | Model D | Model E |
| --- | --- | --- | --- | --- | --- |
| Model A | 98 | 2 | 0 | 0 | 0 |
| Model B | 6 | 94 | 0 | 0 | 0 |
| Model C | 0 | 0 | 99 | 1 | 0 |
| Model D | 0 | 0 | 1 | 57 | 42 |
| Model E | 0 | 0 | 0 | 20 | 80 |

Table S11B

|  | Model D | Model F | Model G |
| --- | --- | --- | --- |
| Model D | 56 | 22 | 22 |
| Model F | 39 | 48 | 13 |
| Model G | 9 | 7 | 84 |

Table S11C

|  | Model A | Model B | Model C | Model D | Model E |
| --- | --- | --- | --- | --- | --- |
| Model A | 65 | 3 | 2 | 15 | 15 |
| Model B | 1 | 99 | 0 | 0 | 0 |
| Model C | 13 | 0 | 95 | 4 | 1 |
| Model D | 11 | 0 | 5 | 38 | 46 |
| Model E | 13 | 1 | 4 | 19 | 63 |

Table S12: Mean posterior probabilities (soft classification) for A) nuclear loci on model set 1 (see Table 2A), B) nuclear loci on model set 2 (see Table 2B) and C) mitochondrial ND2 on model set 1 (see Table 2A). For model specifications, see Table 2 and Fig. 5.

Table S12A

|  | Model A | Model B | Model C | Model D | Model E |
| --- | --- | --- | --- | --- | --- |
| Model A | 0.94 | 0.05 | 0.01 | 0 | 0 |
| Model B | 0.08 | 0.91 | 0 | 0 | 0 |
| Model C | 0 | 0 | 0.98 | 0.01 | 0 |
| Model D | 0 | 0 | 0.01 | 0.53 | 0.46 |
| Model E | 0 | 0 | 0.01 | 0.44 | 0.55 |

Table S12B

|  | Model D | Model F | Model G |
| --- | --- | --- | --- |
| Model D | 0.44 | 0.36 | 0.20 |
| Model F | 0.37 | 0.48 | 0.15 |
| Model G | 0.16 | 0.14 | 0.70 |

Table S12C

|  | Model A | Model B | Model C | Model D | Model E |
| --- | --- | --- | --- | --- | --- |
| Model A | 0.55 | 0.03 | 0.04 | 0.22 | 0.16 |
| Model B | 0.03 | 0.93 | 0.01 | 0.02 | 0.01 |
| Model C | 0.07 | 0.0 | 0.87 | 0.04 | 0.02 |
| Model D | 0.22 | 0.01 | 0.03 | 0.37 | 0.37 |
| Model E | 0.2 | 0.0 | 0.03 | 0.34 | 0.42 |

**REFERENCES**

83. Hackett SJ. Molecular phylogenetics and biogeography of tanagers in the genus *Ramphocelus* (Aves). Mol Phylogenet Evol. 1996; 5(2):368-82.

84. Kirchman JJ, Hackett SJ, Goodman SM, Bates JM. Phylogeny and systematics of ground rollers (Brachypteraciidae) of Madagascar. The Auk 2001;118(4):849-63.

85. Driskell AC, Christidis L: Phylogeny and evolution of the Australo-Papuan honeyeaters (Passeriformes, Meliphagidae). Mol Phylogenet Evol. 2004;31(3):943-60.
