# Supplementary figures and images for: Gene flow during glacial habitat shifts facilitates character displacement in a Neotropical flycatcher radiation
Source: BMC Evol Biol. 2017 Sep 1;17:210. doi: 10.1186/s12862-017-1047-3 (PMC5580441; doi:10.1186/s12862-017-1047-3)

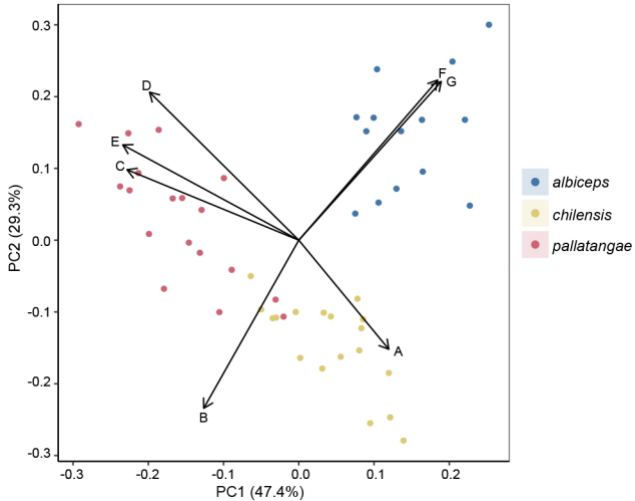

Supplement: Supplementary file 2 — Loadings of the parameters used for principle component analysis of vocal characters (see Fig. 2 for details). Parameters are coded alphabetically. Parameter details are provided in Additional file 1: Table S2. (PDF 289 kb) [file 12862_2017_1047_MOESM2_ESM.pdf]
